# Feasibility of AI-assisted compressed sensing protocols in knee MR imaging: a prospective multi-reader study

**DOI:** 10.1007/s00330-023-09823-6

**Published:** 2023-06-29

**Authors:** Qizheng Wang, Weili Zhao, Xiaoying Xing, Ying Wang, Peijin Xin, Yongye Chen, Yupeng Zhu, Jiajia Xu, Qiang Zhao, Huishu Yuan, Ning Lang

**Affiliations:** Department of Radiology, Peking University Third Hospital, Haidian District, 49 North Garden Road, Beijing, 100191 People’s Republic of China

**Keywords:** Magnetic resonance imaging, Knee, Artificial intelligence, Acceleration, Image enhancement

## Abstract

**Objectives:**

To evaluate the image quality and diagnostic performance of AI-assisted compressed sensing (ACS) accelerated two-dimensional fast spin-echo MRI compared with standard parallel imaging (PI) in clinical 3.0T rapid knee scans.

**Methods:**

This prospective study enrolled 130 consecutive participants between March and September 2022. The MRI scan procedure included one 8.0-min PI protocol and two ACS protocols (3.5 min and 2.0 min). Quantitative image quality assessments were performed by evaluating edge rise distance (ERD) and signal-to-noise ratio (SNR). Shapiro-Wilk tests were performed and investigated by the Friedman test and post hoc analyses. Three radiologists independently evaluated structural disorders for each participant. Fleiss *κ* analysis was used to compare inter-reader and inter-protocol agreements. The diagnostic performance of each protocol was investigated and compared by DeLong’s test. The threshold for statistical significance was set at *p * < 0.05.

**Results:**

A total of 150 knee MRI examinations constituted the study cohort. For the quantitative assessment of four conventional sequences with ACS protocols, SNR improved significantly (*p* < 0.001), and ERD was significantly reduced or equivalent to the PI protocol. For the abnormality evaluated, the intraclass correlation coefficient ranged from moderate to substantial between readers (*κ* = 0.75–0.98) and between protocols (*κ* = 0.73–0.98). For meniscal tears, cruciate ligament tears, and cartilage defects, the diagnostic performance of ACS protocols was considered equivalent to PI protocol (Delong test, *p* > 0.05).

**Conclusions:**

Compared with the conventional PI acquisition, the novel ACS protocol demonstrated superior image quality and was feasible for achieving equivalent detection of structural abnormalities while reducing acquisition time by half.

**Clinical relevance statement:**

Artificial intelligence–assisted compressed sensing (ACS) providing excellent quality and a 75% reduction in scanning time presents significant clinical advantages in improving the efficiency and accessibility of knee MRI for more patients.

**Key Points:**

*• The prospective multi-reader study showed no difference in diagnostic performance between parallel imaging and AI-assisted compression sensing (ACS) was found.*

*• Reduced scan time, sharper delineation, and less noise with ACS reconstruction.*

*• Improved efficiency of the clinical knee MRI examination by the ACS acceleration.*

**Supplementary information:**

The online version contains supplementary material available at 10.1007/s00330-023-09823-6.

## Introduction

Magnetic resonance imaging (MRI) offers outstanding advantages for structural abnormalities of the knee, which is currently one of the most common examinations [1, 2]. A major challenge of MRI acquisition is the prolonged scan duration compared to other modalities, limiting patients’ throughput and potential indications. Longer scan duration would increase the probability of motion artifact interference, especially in patients with knee pain, even resulting in scan failure. Thus, developing advanced accelerated scan protocols is worthy of clinical investigation [3–5].

Artificial intelligence (AI)–based accelerated MRI is in its infancy but has made significant progress in recent years [6]. Although several advanced techniques have been proposed to reduce the image acquisition time, parallel imaging (PI) is proven to reduce the acquisition time while maintaining diagnostic performance and is commercially available for routine clinical use [7, 8]. Increments in the acceleration factor of the conventional acceleration methods may cause reductions in the signal-to-noise ratio (SNR) and increased image artifacts, limiting the feasibility of further accelerated scanning [9, 10]. The recent development of AI-based reconstruction methods pre-learns image structure information from numerous fully sampled MR images via convolutional neural networks and applies the pre-trained knowledge to undersampled image reconstruction, allowing high acceleration levels while maintaining high image quality and potentially yielding a breakthrough in accelerated MRI scanning [6, 11–13].

However, AI-based image reconstruction must be realistic to ensure correct diagnosis in clinical practice. Although some previous studies have investigated the application of AI-based reconstruction, including single sequence studies [14] and retrospective undersampled simulations with accelerated acquisition [15], the main limitation of studies to date is the absence of diagnostic information content [16–18] and surgical ground truth, which limits the support for clinical application [19, 20]. Thus, there are still challenges for AI-based acceleration approaches to enter clinical practice. Further studies are needed to explore the reliability of AI-based acceleration methods in clinical practice.

This study aims at evaluating an AI-assisted compressed sensing (ACS) acceleration technique in the clinical practice of knee imaging, to determine whether ACS protocols can yield accelerated knee MRI scans without compromising the diagnostic value of images and can present advantages in improving the efficiency and accessibility of MRI through improved patient comfort and fewer motion artifacts. We also evaluated a 2.0-min accelerated protocol, which may hold promise for rapid knee scans of patients with special clinical needs.

## Materials and methods

### Participants

**Fig. 1:**
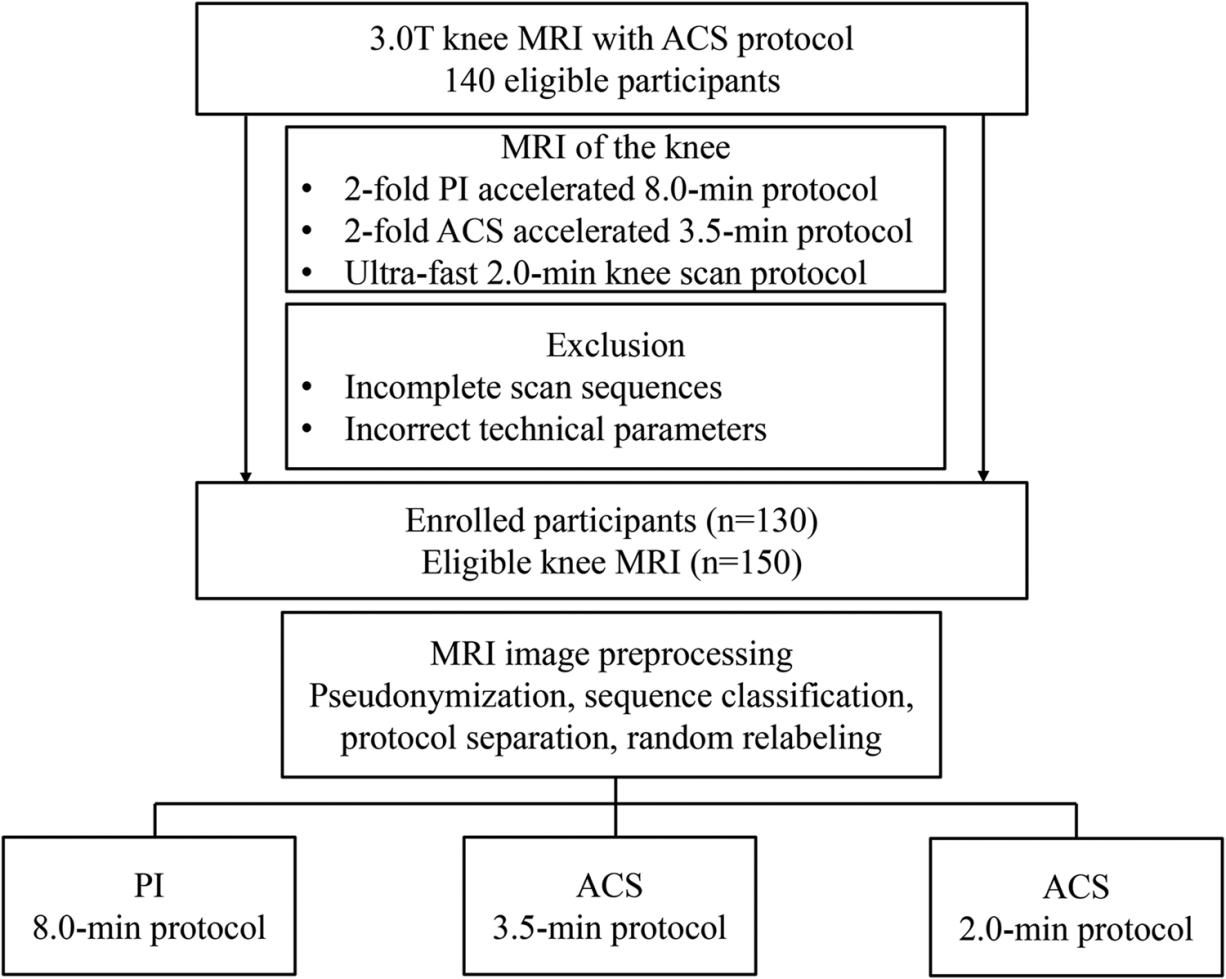
Flowchart of the study. PI, parallel imaging; ACS, artificial intelligence–assisted compressed sensing

A total of 150 knee MRI images from 130 participants with 3.0 T knee MRI scans with ACS protocols in our institution from March to September 2022 were eligible for this study. Ten ineligible scans were excluded (six scans with incomplete sequences and four scans using incorrect technical parameters). We did not exclude participants with knee arthroscopic surgery history unless severe implant artifacts. Eight participants with previous anterior cruciate ligament replacement were included in the study. The study workflow is shown in Fig. 1.

This study was approved by our Institutional Research Ethics Board (No. M2022394) and adhered to the tenets of the Declaration of Helsinki. Details of the scanning parameters were communicated to all participants, and written informed consent was obtained.

### MRI protocols

All participants underwent MRI acquisition using a 3.0-T MRI scanner (uMR 880, United Imaging) with a 12-channel receiver knee coil. Our institution’s routine knee MRI protocol included two-dimensional sagittal, coronal, and axial proton density fat-saturated (PDFS) fast spin-echo (FSE) sequences and sagittal T1-weighted FSE sequences. After the conventional four sequences of PI protocol, two ACS four-sequence scanning protocols (ACS 3.5-min protocol and ACS 2.0-min protocol) were followed. All scanning parameters are listed in Table S1.

### ACS image reconstruction

The ACS technology is an FDA-approved method for accelerating image acquisition using convolutional neural networks (CNN). While CNN-based methods have shown superior reconstruction quality, their performance and reliability in clinical settings are often uncertain due to the black-box nature of the network. To address this uncertainty, ACS integrates the output of the trained AI module as an additional constraint into the compressed sensing framework. This is achieved by adding a regularization term based on the difference between the reconstructed image and the predicted image of the AI module, as indicated in the equation [21]. The ACS network was trained using a dataset of two million fully sampled images previously acquired from phantoms (2%) and human volunteers (98%).

Meanwhile, the architectural design employed in the iteration processes was derived from the k-space, with multiscale sparsification integrated. Compressed sensing, partial Fourier, and parallel imaging are all incorporated into the mathematical model. Simulation tests have demonstrated that ACS is able to correct errors in the output generated by the AI model and achieve high consistency compared to the fully sampled golden standard [21]. Further details about model training can be found in the supplementary.

$${\text{argmin}}_{x}{\left|\left| Ex-y \right|\right|}_{2}^{2}+{\lambda }_{1}{\left|\left| \psi \left(x\right)\right|\right|}_{1}+{\lambda }_{2}{\left|\left| \phi \left({x}_{AI},x\right)\right|\right|}_{w}+{\lambda }_{3}{\left|\left| PI \right|\right|}_{m}+{\lambda }_{4}{\left|\left| PF \right|\right|}_{n}$$

### Image pre-processing

Images of each participant were desensitized and classified by the 8.0-min PI, 3.5-min ACS, and 2.0-min ACS protocols, and the sequence names were invisible to readers. All 450 knee MRI images from the three MRI protocols were reviewed by two raters (Q.W. and Q.Z.) using a 5-point Likert scale (range: 1 = “worst” to 5 = “best”). All images had a Likert score of at least 4 and were considered appropriate for diagnosis. Overall image quality for both PI and ACS was rated excellent (median, 5; interquartile range, 4–5).

### Quantitative analysis

Image sharpness was measured by determining the edge rise distance (ERD; the distance between points at 10% and 90% of the maximum intensity value) [14, 22–25]. Lower ERD values indicated better sharpness. After image normalization, five radiologists measured ERD in each sequence at the interface between the bone cortex and marrow (Q.W., P.X., Y.C., Z.Z., and Q.Z.) with 4–8 years’ experience using ImageJ software (https://imagej.nih.gov/ij/). The particle analysis tool (Plot Profile) was used to generate the profile curves. The region from the bone cortex to bone marrow was easily accessible in all sequences and less susceptible to interference.

**Fig. 2:**
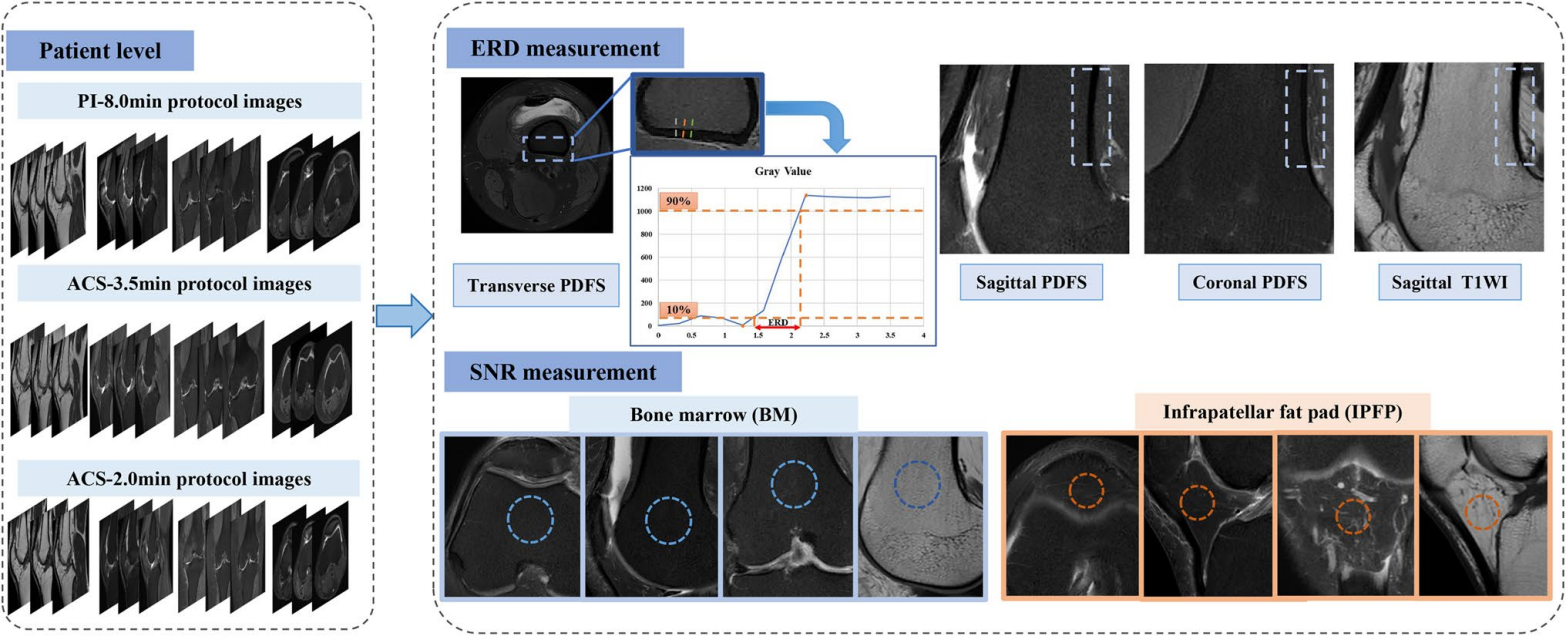
Quantitative evaluation method diagram. PI, parallel imaging; ACS, artificial intelligence–assisted compressed sensing; ERD, edge rise distance; PDFS, proton density–weighted fat-suppressed; SNR, signal-to-noise ratio

Quantitative SNR assessments were performed manually using two circular ROIs for femoral bone marrow (BM) and infrapatellar fat pad (IPFP) on one slice at identical levels, avoiding abnormal signal lesions. Figure 2 demonstrates ROI examples and the measurement methods for each sequence.

### Reader assessments

Three radiologists performed all images for abnormality diagnosis with 8 (Y.W.), 10 (X.X.), and 15 (N.L.) years of experience in musculoskeletal MRI diagnosis. A high PDFS signal that breached the meniscal surface indicated meniscal tears. For the ligaments, fiber discontinuity of 50% or more suggested tears. Bone marrow edema and cartilage defects were evaluated at the patella, femoral trochlea, and tibial plateau, and defects larger than 1/3rd of the area were considered cartilage defects. Before formal assessments, training was conducted using 20 external cases to ensure consistent criteria by the three readers. Interpretations with different protocols for each patient were spaced 4 weeks apart to limit the possibility of recall bias.

All readers performed evaluations independently and were blinded to the assessments of other readers. All results were collected by a rater (Q.W.), and consensus was obtained according to the following criteria: consensus of abnormality was determined by the majority conclusions of three radiologists for each patient; if there is a complete disagreement between the three radiologists, the case will be discussed again until an agreement is reached. The discussion was only to get a consensus without affecting the previous results of each radiologist, and changes were not allowed. For participants who underwent surgery (*n* = 44), findings at arthroscopic knee surgery were used as the reference standard.

### Statistical analysis

Fleiss *κ* analysis was used to compare inter-reader and intra-method agreement for the three knee MRI protocols. The *κ* statistic was interpreted as follows: less than 0.20, poor agreement; 0.21–0.40, fair agreement; 0.41–0.60, moderate agreement; 0.61–0.80, substantial agreement; and 0.81–1.00, excellent agreement. The diagnostic performance, sensitivity, specificity, accuracy, and receiver operating curve (ROC) of the MRI protocols were derived using the consensus of three readers, with arthroscopic surgical reports serving as the reference standard. The area under the curve (AUC) with the Delong method of MedCalc Statistical Software (MedCalc Software Ltd, version 20.022) was used. Shapiro-Wilk tests were performed and investigated by the Friedman test and post hoc analyses using SPSS software (IBM SPSS Statistics for Windows, Version 24.0). The threshold for statistical significance was set at *p*<0.05.

## Results

### Participant characteristics

The study included 150 knee MRI scans (left:right = 66:84) representing chronic knee symptoms (97, 65%), acute knee trauma (45, 30%), and postoperative implants (8, 5%) in 130 participants (mean age, 39.8 years; range, 8.0–77.0 years). The characteristics of the participants are shown in Table 1.

**Table 1:** Participants demographics and clinical information

Characteristics	Study cohort (*n* = 130/150)
Age (y)	39.8 ± 14.3
Sex	
Female	63
Male	67
Laterality	
Right knee	84
Left knee	66
Major knee symptoms	
Acute trauma	45
Chronic pain	97
Postoperative follow-up	8

Data are expressed as means ± standard deviations or as counts with percentages in parentheses

### Comparison of quantitative assessments

**Fig. 3:**
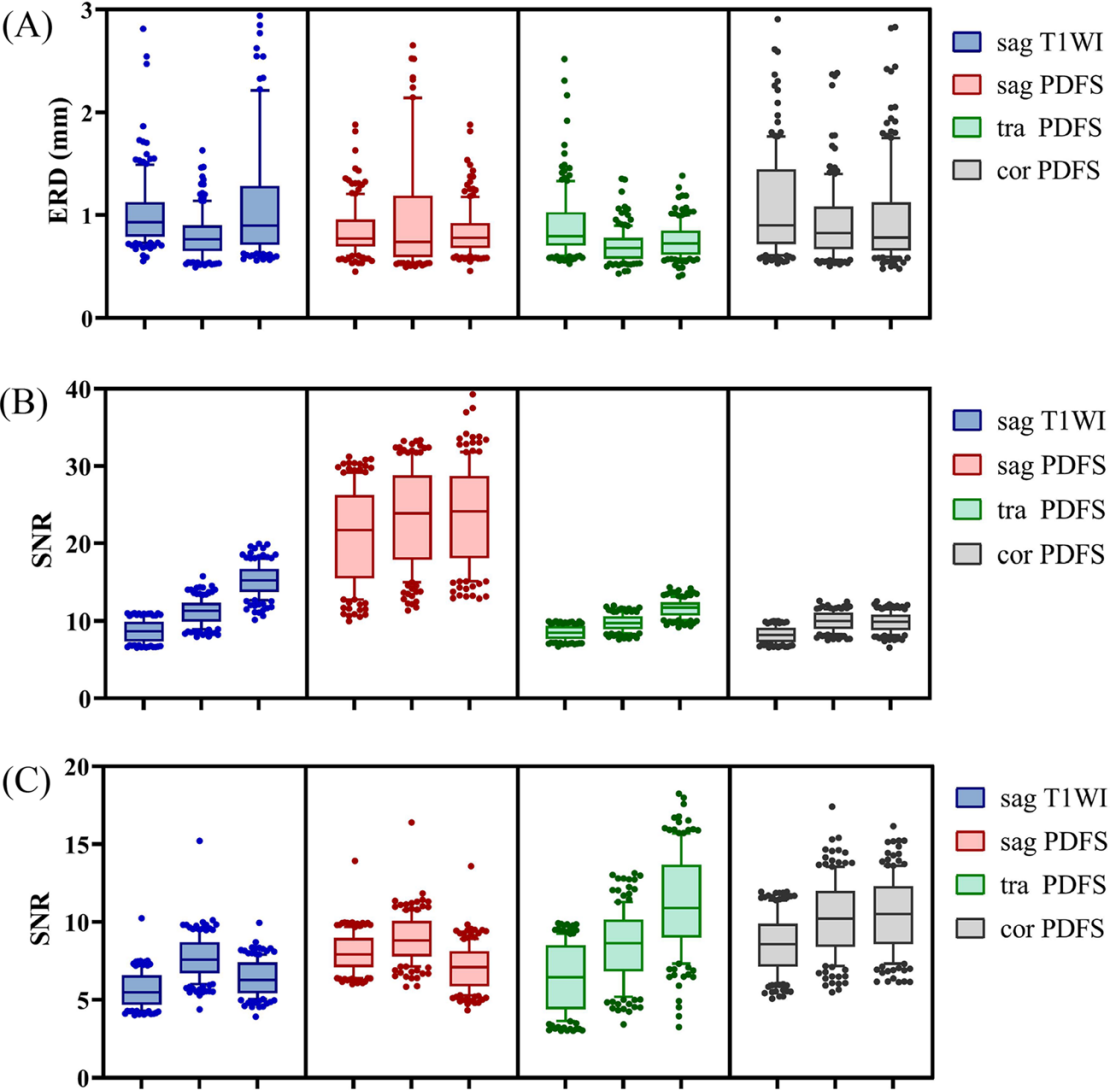
Comparison of quantitative assessments. The graphs show the values of edge rise distance (**A**) and signal-to-noise ratio (**B**, **C**) between different protocols. Boxes of the same color from left to right for 8.0-min PI protocol, 3.5-min ACS protocol, and 2.0-min ACS protocol, respectively. PI, parallel imaging; ACS, artificial intelligence–assisted compressed sensing

In assessments of image sharpness, both 3.5- and 2.0-min ACS protocols showed reduced ERD values compared with the PI protocol in the sagittal T1WI (*p* < 0.001), transverse (*p* < 0.001), and coronal (*p* = 0.002) PDFS sequences. Despite the reduced values in ACS protocols, ERD was not significantly different (*p*=0.2) among the 3.5-min ACS (1.09 ± 0.74), 2.0-min ACS (0.83 ± 0.78), and PI (0.85 ± 0.77) protocols in sagittal PDFS sequence. Although the SNR values for the two ROIs were not identical and the SNR for the bone marrow was higher, ACS protocols were superior to the conventional PI protocol in all four sequences (*p* < 0.001) in both ROIs (BM and IPFP). The combination of quantitative measurements of ERD and SNR shows that the ACS protocol scans yield better image quality. The corresponding results are shown in Fig. 3, and the detailed results are in Supplementary Tables S2–S5.

### Inter-reader agreement and inter-method reliability

Table 2 presents the inter-reader agreement for diagnosing knee abnormalities using the ACS and PI protocols. All three protocols showed substantial-to-excellent agreement (min: 0.75, max: 0.98) for the diagnostic performance of abnormality. For the diagnosis of meniscal tears (medial/lateral), the inter-reader agreement was excellent for interpretations with the 8.0-min PI (*κ* = 0.901/0.869), 3.5-min ACS (*κ* = 0.883/0.869), and 2.0-min ACS (*κ* = 0.897/0.802) protocols. Inter-reader agreement ranged from substantial to excellent for the diagnosis of cruciate ligament tears, with a minimum *κ* value of 0.75. All three protocols showed high inter-reader agreement to detect bone marrow edema and cartilage defects (*κ* > 0.80). The ACS 2.0-min protocol detected fewer ranges and numbers of structural abnormalities than the 8.0-min PI and 3.5-min ACS protocols. The three scanning protocols are almost consistent in fracture detection. The results of the inter-reader agreement for different scanning protocols are shown in Table 2.

**Table 2:** Inter-reader agreement for different scanning protocols

Structural disorders and protocols	Number of lesions detected by observers				Inter-reader agreement
	Reader 1	Reader 2	Reader 3	Consensus	
Medial meniscus tears					
PI protocol	41	38	42	38	0.901
ACS 3.5-min protocol	43	39	37	37	0.883
ACS 2-min protocol	40	38	38	38	0.897
Lateral meniscus tears					
PI protocol	16	10	12	10	0.869
ACS 3.5-min protocol	19	12	13	12	0.849
ACS 2-min protocol	17	15	12	12	0.802
Anterior cruciate ligament tear					
PI protocol	14	20	19	14	0.854
ACS 3.5-min protocol	12	21	15	12	0.752
ACS 2-min protocol	13	13	14	13	0.927
Posterior cruciate ligament tears					
PI protocol	2	2	2	2	0.855
ACS 3.5-min protocol	2	2	2	2	0.880
ACS 2-min protocol	2	4	2	2	0.796
Medial collateral ligament tears					
PI protocol	5	7	8	5	0.869
ACS 3.5-min protocol	3	7	6	3	0.786
ACS 2-min protocol	3	3	6	3	0.899
Bone marrow edema					
PI protocol	110	101	109	101	0.879
Patella	38	38	38	38	
Femoral trochlea	34	31	34	31	
Tibial plateau	38	32	37	32	
ACS 3.5-min protocol	113	111	111	110	0.967
Patella	41	38	40	40	
Femoral trochlea	34	34	35	34	
Tibial plateau	38	39	36	36	
ACS 2-min protocol	101	110	108	101	0.805
Patella	36	38	36	36	
Femoral trochlea	31	34	37	31	
Tibial plateau	34	38	35	34	
Cartilage defects					
PI protocol	90	85	91	84	0.932
Patella	40	41	44	40	
Femoral trochlea	37	31	34	31	
Tibial plateau	13	13	13	13	
ACS 3.5-min protocol	91	95	92	83	0.840
Patella	40	46	45	40	
Femoral trochlea	38	30	33	30	
Tibial plateau	13	19	14	13	
ACS 2-min protocol	85	84	87	82	0.826
Patella	41	43	42	41	
Femoral trochlea	31	30	34	30	
Tibial plateau	13	11	11	11	
Fracture					
PI protocol	8	8	7	7	0.984
ACS 3.5-min protocol	8	7	7	7	0.947
ACS 2-min protocol	7	8	8	7	0.950

*PI*, parallel imaging; *ACS*, artificial intelligence–assisted compressed sensing

Inter-method reliability was calculated for each reader, which showed that the agreement between PI and the ACS protocols ranged from substantial to excellent (reader1: *κ* = 0.80–1.00; reader2: *κ* = 0.73–0.98; reader3: *κ* = 0.78–0.98). Agreement among protocols improved in consensus among readers, with all protocols showing excellent agreement (*κ* = 0.84–0.98). The results of the inter-protocol agreement are shown in Table 3.

**Table 3:** Inter-protocol agreement of the three readers

Abnormality	Reader 1	Reader 2	Reader 3	Consensus
Medial meniscus tears	0.96 (0.95–0.97)	0.98 (0.97–0.99)	0.97 (0.96–0.98)	0.98 (0.97–0.99)
Lateral meniscus tears	0.96 (0.95–0.97)	0.73 (0.64–0.80)	0.88 (0.84–0.91)	0.90 (0.86–0.92)
Anterior cruciate ligament tears	0.96 (0.95–0.97)	0.84 (0.78–0.88)	0.78 (0.71–0.83)	0.87 (0.83–0.90)
Posterior cruciate ligament tears	1.00 (1.00–1.00)	0.86 (0.81–0.89)	0.90 (0.87–0.92)	0.90 (0.87–0.92)
Medial collateral ligament tears	0.93 (0.91–0.95)	0.76 (0.69–0.82)	0.96 (0.95–0.97)	0.84 (0.79–0.88)
Bone marrow edema	0.80 (0.73–0.85)	0.87 (0.84–0.91)	0.91 (0.88–0.93)	0.93 (0.90–0.95)
Cartilage defects	0.97 (0.96–0.98)	0.78 (0.71–0.84)	0.91 (0.88–0.93)	0.91 (0.88–0.93)
Fractures	0.93 (0.91–0.95)	0.98 (0.98–0.99)	0.98 (0.98–0.99)	0.98 (0.98–0.99)

### Diagnostic performance

**Fig. 4:**
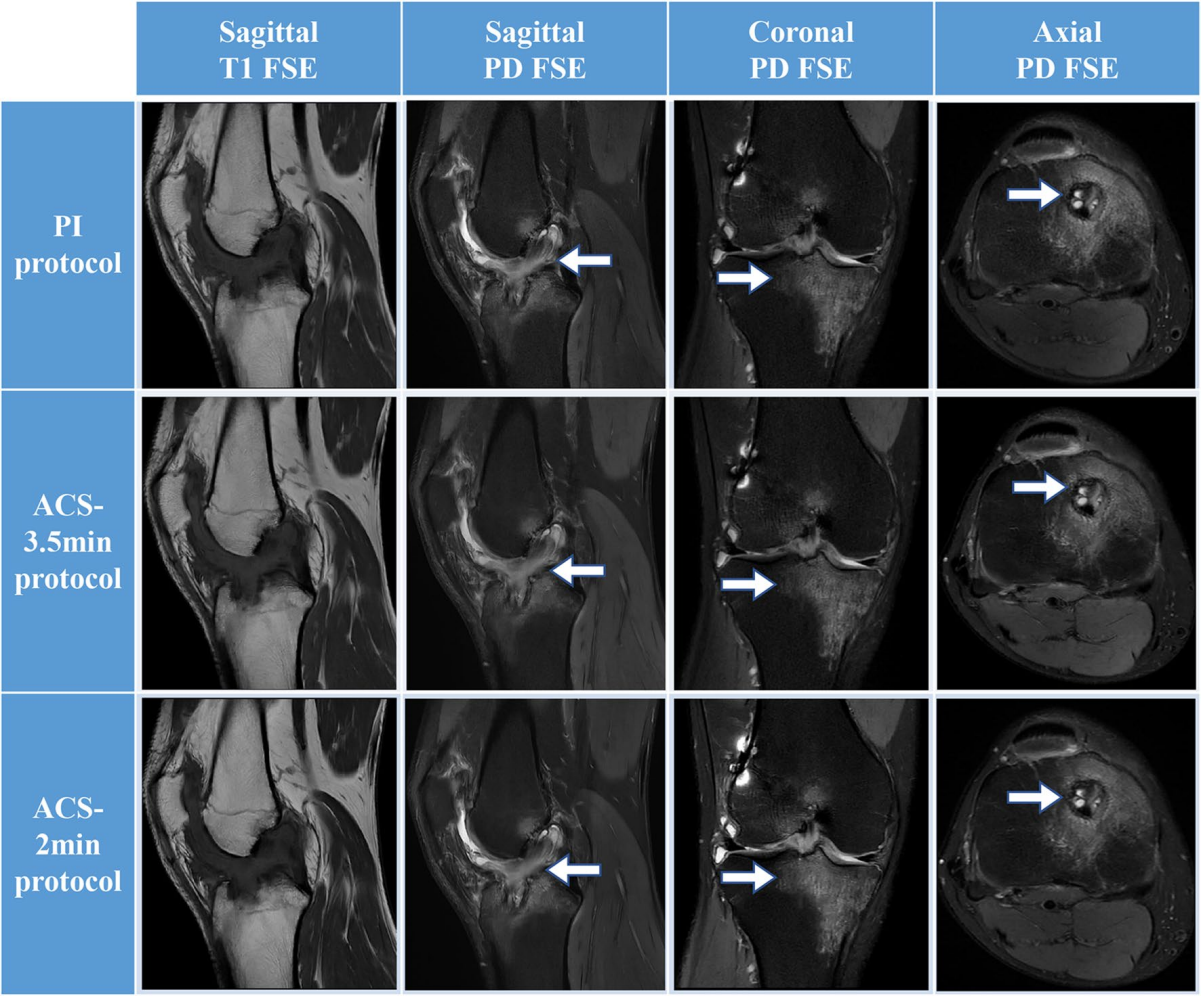
MRI images of a 23-year-old male after anterior cruciate ligament (ACL) reconstruction. From left to right, sagittal T1-weighted images, proton density–weighted fat-suppressed (PDFS) sagittal, coronal, and axial images of fast spin-echo (FSE) imaging, respectively. T1-weighted images show bone condition; sagittal PDFS shows ACL continuity (arrows); coronal PDFS shows bone marrow edema (arrows); transverse PDFS shows bone tract and bone marrow edema of the tibia (arrows)

**Fig. 5:**
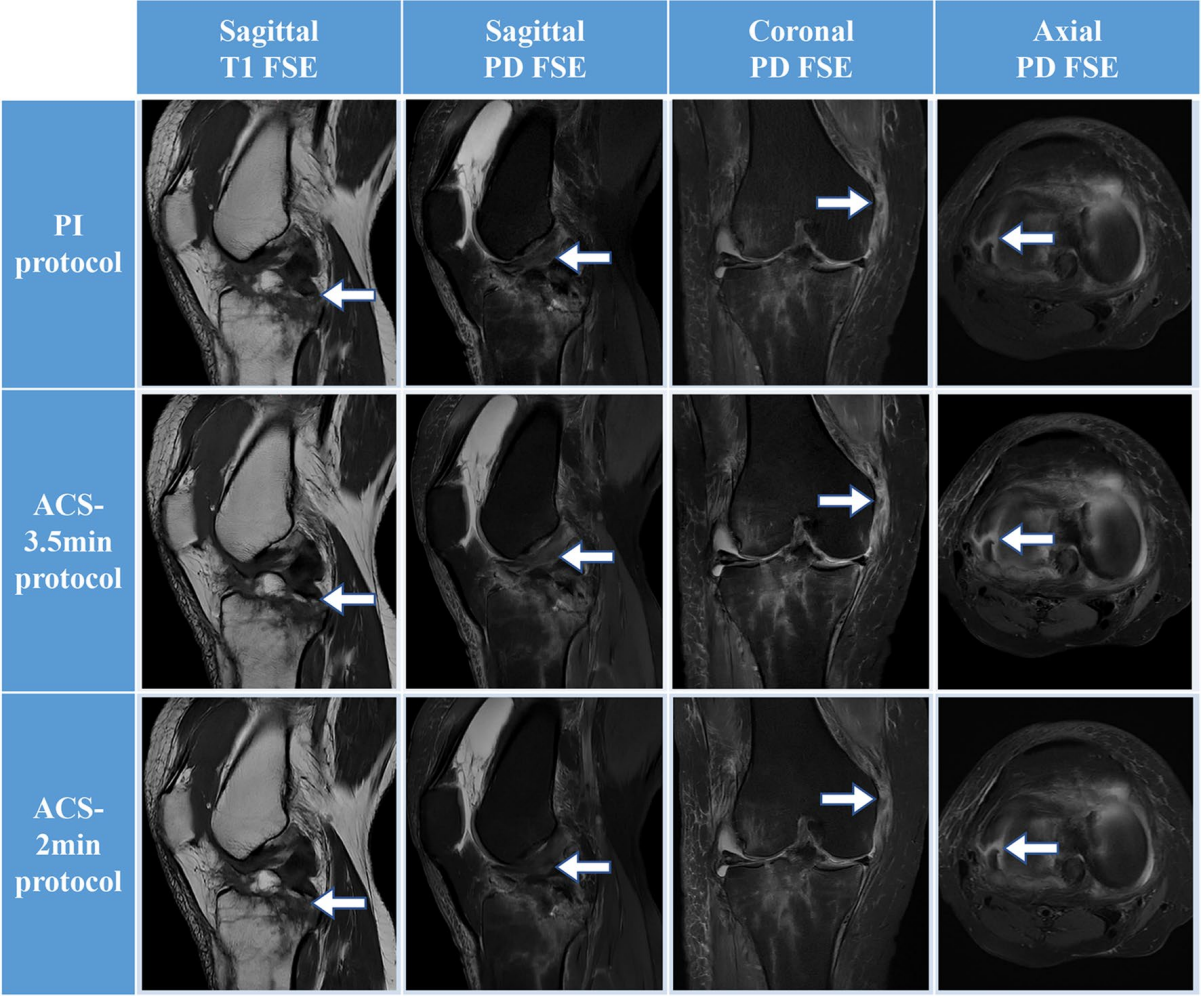
MRI images of a 50-year-old woman with acute trauma. From left to right, sagittal T1-weighted, proton density (PD)–weighted fat-suppressed sagittal, coronal, and axial images of fast spin-echo (FSE) imaging, respectively. Sagittal T1 image shows tibial plateau fracture (arrows); sagittal PD image shows anterior cruciate ligament injury (arrows) and bone marrow edema, joint cavity effusion, and synovial hyperplasia; coronal PD image shows medial collateral ligament tear (arrows); transverse axial view shows medial meniscus tear (arrows)

**Fig. 6:**
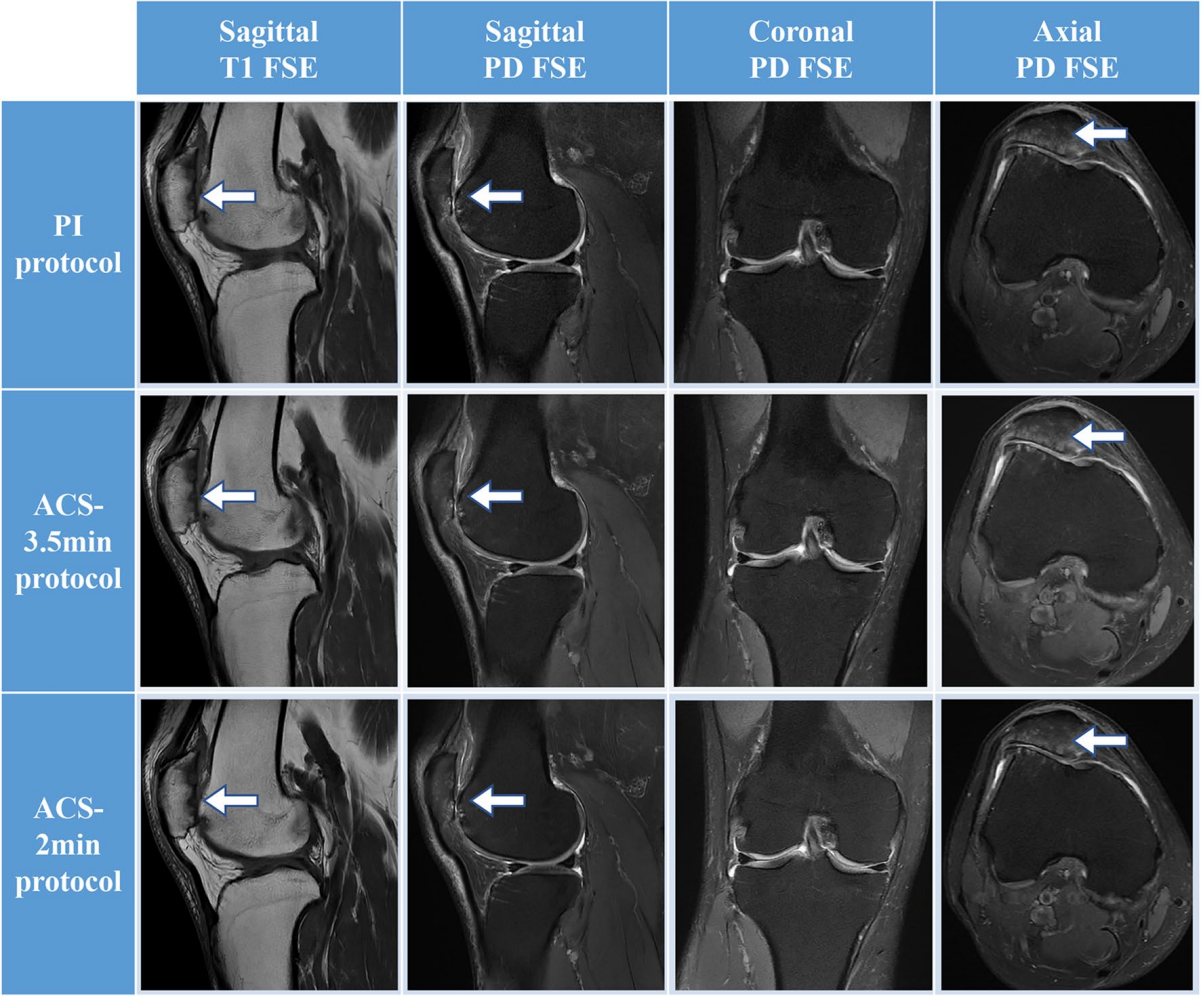
MRI images of a 44-year-old male athlete with patellofemoral arthropathy. From left to right, sagittal T1-weighted images, proton density–weighted fat-suppressed (PDFS) sagittal, coronal, and axial images of fast spin-echo (FSE) imaging, respectively. Patellofemoral and femoral talocrural articular cartilage and subchondral bone damage (arrows) are clearly demonstrated on sagittal T1, sagittal, and transverse PDFS images. The meniscus is intact on coronal PDFS images

In this study, 44 of 130 participants underwent surgery in our institution with available surgical records. Arthroscopic reports of structural abnormalities were used as the gold standard to assess the diagnostic performance of three scanning protocols. The overall sensitivity of MRI evaluation of meniscal tears (medial/lateral) compared with arthroscopic diagnosis results was 95.7%/84.6%, 82.6%/84.6%, and 82.6%/96.8% in 8.0-min PI, 3.5-min ACS, and 2.0-min ACS, respectively, and the specificity of all three protocols was 100%. The real anterior cruciate ligament tears were detected, and the PI protocol is more sensitive (93.3%) than the ACS protocols (3.5-min ACS: 86.7%, 2.0-min ACS: 73.3%) but less specific (89.7%) than the ACS protocols (3.5-min ACS: 100%, 2.0-min ACS: 96.6%). For cartilage defects, the difference in diagnosis performance (sensitivity/specificity) between the three protocols was insignificant (8.0-min PI: 92.0%/78.9%; 3.5-min ACS: 88.0%/84.2%, 2.0-min ACS: 88.0%/84.2%). The difference in the areas between the PI and ACS evaluation ROC was not statistically significant (*p* > 0.05) by the Delong test in any structural abnormality. The results show that the ACS method achieves equivalent diagnostic reliability to PI for detecting knee abnormalities (Table 4). Image examples of PI and ACS are provided in Figs. 4, 5 and 6.

**Table 4:** Diagnostic performance of three different scanning protocols

Abnormality and protocol	TP	FN	TN	FP	Sensitivity (%)	Specificity (%)	AUC	SE	95% CI	Difference	*p* value
Medial meniscus tears											
PI protocol	22	1	21	0	95.7	100	0.978	0.0217	0.88–1.00	-	-
ACS 3.5-min protocol	19	4	21	0	82.6	100	0.913	0.0404	0.79–0.98	0.07	0.17
ACS 2-min protocol	19	4	21	0	82.6	100	0.913	0.0404	0.79–0.98	0.07	0.17
Lateral meniscus tears											
PI protocol	11	2	31	0	84.6	100	0.923	0.0521	0.80–0.98	-	-
ACS 3.5-min protocol	11	2	31	0	84.6	100	0.923	0.0521	0.80–0.98	0.00	> 0.99
ACS 2-min protocol	10	3	30	1	96.8	100	0.868	0.0629	0.73–0.95	0.05	0.19
Anterior cruciate ligament tears											
PI protocol	14	1	26	3	93.3	89.7	0.915	0.044	0.79–0.98	-	-
ACS 3.5-min protocol	13	2	29	0	86.7	100	0.933	0.0454	0.82–0.99	0.02	0.78
ACS 2-min protocol	11	4	28	1	73.3	96.6	0.849	0.0616	0.71–0.94	0.07	0.41
Cartilage defect											
PI protocol	23	2	15	4	92.0	78.9	0.855	0.0555	0.72–0.94	-	-
ACS 3.5-min protocol	22	3	16	3	88.0	84.2	0.861	0.0543	0.72–0.95	0.01	0.93
ACS 2-min protocol	22	3	16	3	88.0	84.2	0.861	0.0543	0.72–0.95	0.01	0.93

*TP*, true-positive; *FN*, false-negative; *TN*, true-negative; *FP*, false-positivse; *AUC*, area under the curve; *SE*, standard error; *CI*, confidence interval; *PI*, parallel imaging; *ACS*, artificial intelligence–assisted compressed sensing

## Discussion

Our study showed that artificial intelligence–assisted compressed sensing (ACS) can be used for reconstruction in knee MRI and performed as well with conventional 2-fold parallel imaging (PI) to detect internal knee derangement while reducing the acquisition time by even more than 70%. Moreover, ACS protocols showed good-to-excellent inter-reader and inter-method agreement with an improved signal-to-noise ratio (SNR) and reduced edge rise distance (ERD).

MRI is one of the most dynamic and safe imaging techniques in clinical practice today and offers an unparalleled soft tissue resolution. However, MRI is an inherently slow imaging modality, limited by traditional Nyquist sampling requirements, and prone to motion artifacts. With the increasing demand in clinical practice, acceleration of image data acquisition has been a focus of research, yielding several undersampling reconstruction techniques such as partial Fourier (PF), PI, simultaneous multislice (SMS) imaging, compressed sensing (CS), and AI-based reconstruction algorithms. Despite its wide clinical usage and integration into commercial MR systems, PI with multiple receiver coils typically requires limiting the acceleration factor to 2 while maintaining spatial resolution [9]. Similarly, the acceleration factor in SMS is generally limited to 4 due to slice crosstalk artifacts and increased energy deposition into the tissue through multislice excitation.

In contrast, undersampled reconstruction techniques such as CS can accelerate images beyond a factor of 4. The assumption underlying CS is that the k-space data are randomly undersampled. CS-based reconstruction techniques employ pseudorandom trajectories along with one or several sparse transforms such as finite differences or wavelet operators [26]. After receiving Food and Drug Administration approval in 2017, CS-based reconstruction is being gradually adopted into clinical practice. Convolutional neural network–based ACS acceleration technology will likely be the next entry point for accelerated imaging [13]. However, incorporating such protocols into clinical practice will require ongoing and extensive validation of deep learning models and a thorough understanding of their generalizability and limitations.

Musculoskeletal imaging is highly demanding regarding resolution, clarity, and image quality. However, patients with pain or a limited range of motion may experience difficulty in maintaining stable imaging positions. In our study, ACS technology showed clinical utility in accelerated knee MRI scan acquisition, which has been previously assessed in abdomen imaging of the liver [27]. The artificial intelligence–based filtering and interpolation image reconstruction techniques are based on coding and decoding convolutional neural networks to achieve high-resolution imaging without loss of time or image SNR by deep learning to remove noise for better image quality [12]. Our study included three accelerated scanning protocols based on two different technologies: the PI 8-min protocol and the ACS 3.5- and 2.0-min protocols. Image evaluation was performed by assessing image quality and diagnostic efficacy. The comparison of quantitative assessments and task-based (diagnostic) performance metrics showed that ACS-accelerated MRI scans are equal to or close to conventional PI protocols in diagnosing most structural knee disorders with reduced noise and significantly improved image sharpness and SNR. This study included a diverse range of patients with acute trauma, chronic injury, cruciate ligament reconstruction, and post-arthroscopic surgery, enhancing the representativeness of its findings for clinical practice.

Our results showed that the ACS technique can be effectively used to reconstruct two-dimensional acquired MR images, as shown in this study, and that the process is fast or negligible without obstacles in clinical application with extremely short or negligible processing times. Advanced technologies can be used in combination to create synergies in practice, and the ACS 2.0-min protocol in this study is a hybrid acceleration ultra-fast scanning protocol. Similar hybrid acceleration techniques are available with several vendors, and our study shows that the ability of such methods to diagnose most structural disorders of the knee is similar to the conventional PI protocol. However, for the detection of cartilage defects, despite maintaining higher reader consistency (*κ* = 0.840), the lesion extent was underestimated, which may be due to the increased layer thickness (4 mm). Nevertheless, rapid scans are essential under specific examination constraints, such as difficulties in patient cooperation (knee pain, pediatric patients, trauma screenings, etc.). The ACS 2.0-min protocol proposed in this study may help solve these problems by rapidly yielding low-cost, ionizing radiation-free MRI scans with the potential to replace radiography.

Although AI reconstruction of MR images has been investigated previously, those studies compared sequence equivalence on single sagittal sequences of the knee [14] or lacked the diagnostic accuracy of the protocols [15, 19, 20]. To the best of our knowledge, this is the first study to compare ACS-accelerated protocols with a standard clinical MRI protocol in all four sequences (one T1-weighted FSE and three proton density–weighted FSE sequences with fat saturation) with the diagnostic accuracy of the protocols compared. Moreover, according to the indications of knee MRI application in actual clinical practice, the types of participants included in this study were more diverse than in previous studies. Participants after cruciate ligament surgery were included. Interobserver agreement was good for all three protocols, suggesting that ACS-accelerated MRI did not introduce more artifacts interfering with the observation of knees with certain metal implants and can be used for postoperative examinations. In addition, the absence of radiation makes knee MRI especially applicable in children with joint discomfort; thus, pediatric participants were included in this study for the examination.

Although the findings demonstrated the feasibility of the proposed ACS method, this study had some limitations. First, all images were obtained from a 3.0-T MRI scanner from a single vendor, and no validation was performed on other machines. Further studies at multiple vendors and other scanning sites are warranted to assess the scalability of AI-assisted acceleration technology fully. Second, only a small percentage of participants underwent arthroscopy and had pathological findings for diagnostic performance analysis, potentially diminishing the persuasiveness of the results. However, in clinical practice, not all patients with the detected disease require arthroscopy, and interobserver and inter-method agreements may address this concern. Third, the low incidence of some intra-articular knee lesions in our study cohort may have limited statistical efficacy. Necessary future steps include correlating readers’ assessments of pathological findings compared against ground truths (arthroscopy) and establishing robustness across the various scanners and vendors. Thus, additional research with a larger pool of patients from internal and multi-center studies is necessary to verify these findings. Further studies would also include validating the ACS acceleration technique on quantitative MRI, such as T1/T2 mappings and 3D sequences.

In conclusion, comparing the artificial intelligence–assisted compressed sensing (ACS) technique with parallel imaging (conventional acceleration factor of 2.0) illustrated that ACS acceleration methods using novel reconstruction algorithms can reduce scanning times and maintain high image quality and equivalent diagnostic performance.

### Supplementary Information

Below is the link to the electronic supplementary material.

Supplementary file1 (PDF 267 KB)

## Abbreviations

ACS: AI-assisted compressed sensing
AI: Artificial intelligence
CS: Compressed sensing
ERD: Edge rise distance
FSE: Fast spin-echo
ICC: Intraclass correlation coefficient
PDFS: Proton density–weighted fat-suppressed
PI: Parallel imaging
SNR: Signal-to-noise ratio

## Declarations

### Guarantor

The scientific guarantor of this publication is Ning Lang.

### Statistics and biometry

Nan Li (Peking University Third Hospital, Research Center of Clinical Epidemiology) kindly provided statistical advice for this manuscript.

### Study subjects or cohorts overlap

None of our study subjects or cohorts has been previously reported.

### Methodology

• prospective

• diagnostic study

• performed at one institution
