# Supplementary material for: Feasibility of AI-assisted compressed sensing protocols in knee MR imaging: a prospective multi-reader study
Source: Eur Radiol. 2023 Jun 29;33(12):8585–96. doi: 10.1007/s00330-023-09823-6 (PMC10667384; doi:10.1007/s00330-023-09823-6)
Supplement: Supplementary file 1 — Supplementary file1 (PDF 267 KB) [file 330_2023_9823_MOESM1_ESM.pdf]

Supplementary

ACS image reconstruction

The architecture of the convolutional neural networks used for image reconstruction in this study was extended with paired undersampled and full-sampled images. The network used in this study is similar to U-net, with a residual block consisting of two convolution operations and a jump connection replacing the convolution operation in the original U-net. To speed up the learning process, a long jump connection was added to learn the residuals between fully sampled and undersampled images. To further improve the quality of the reconstructed images, a least-squares generative adversarial network training technique was used [1; 2]. In previous studies, we applied this technology in single-breath-hold T2WI liver MRI [3], and the same network structure of the deep learning-based fast MRI reconstruction framework was used in this study.

Reference:

1 Quan TM, Nguyen-Duc T, Jeong WK (2018) Compressed Sensing MRI Reconstruction Using a Generative Adversarial Network With a Cyclic Loss. IEEE Trans Med Imaging 37:1488-1497

2 Yang G, Yu S, Dong H et al (2018) DAGAN: Deep De-Aliasing Generative Adversarial Networks for Fast Compressed Sensing MRI Reconstruction. IEEE Trans Med Imaging 37:1310-1321

3 Sheng RF, Zheng LY, Jin KP et al (2021) Single-breath-hold T2WI liver MRI with deep learning-based reconstruction: A clinical feasibility study in comparison to conventional multi-breath-hold T2WI liver MRI. Magn Reson Imaging 81:75-81

Table S1. MRI sequence parameters used in three protocols

Parameter \ Protocol	PI protocol				ACS-3.5min protocol				ACS-2min protocol			
	Sagittal T1 FSE	Sagittal PD FSE	Coronal PD FSE	Transverse PD FSE	Sagittal T1 FSE	Sagittal PD FSE	Coronal PD FSE	Transverse PD FSE	Sagittal T1 FSE	Sagittal PD FSE	Coronal PD FSE	Transverse PD FSE
Repetition time, ms	600	2600	2600	2600	600	2750	2400	2400	400	2450	1900	2600
Echo time, ms	8.9	37.3	37.3	37.3	8.5	40.0	37.3	37.3	6.2	50.3	43.0	46.6
Echo train length	3	6	6	6	4	10	7	7	5	12	10	11
No. excitations	1	1	1	1.5	1	1	1	1	1	1	1	1
Bend width,Hz	250	180	180	180	220	245	185	185	400	220	300	250
Flip angle, degrees	90	90	90	90	90	90	90	90	90	90	90	90
Field of view, mm	160×160	160×160	160×160	160×160	160×160	160×160	160×160	160×160	160×160	160×160	160×160	160×160
Matrix	252×336	272×320	272×320	272×320	252×336	272×320	272×320	272×320	252×336	272×320	272×320	272×320
Slice thickness/gap, mm	3.5/0.5	3.5/0.5	3.5/0.5	4.0/0.5	3.5/0.5	3.5/0.5	3.5/0.5	4.0/0.5	4.0/0.5	4.0/0.5	4.0/0.5	4.0/0.5
Concatenation	3	1	1	1	2	1	1	1	2	1	1	1
Acceleration factor	2	2	2	2	2	2	4	4	3	1	1	2
Mean acquisition time, min:s	1:39	1:54	1:24	1:45	0:35	0:58	0:34	0:46	0:23	0:40	0:30	0:24

Note: Multiple values indicate parameter differences between scan sequences. Sagittal, coronal, and axial T2 fat-suppression sequences and T1WI sagittal scan parameters of each scanning protocol, respectively.

Table S2. Quantitative evaluation of edge rise distance (ERD) and signal-to-noise ratio (SNR) in sagittal T1WI images

Variable and protocol		Mean	Median	SD	IQR	*P* value for Friedman test	*P* value for post hoc analysis after Friedman test		
							PI	ACS-3.5min	ACS-2min
ERD									
	PI	1.03	0.93	0.41	0.33		-	<.001	0.676
	ACS-3.5min	0.80	0.76	0.22	0.24	<.001	<.001	-	<.001
	ACS-2min	1.16	0.90	0.73	0.57		0.676	<.001	-
SNR-Bone									
	PI	21.05	21.71	6.0	10.53		-	<.001	<.001
	ACS-3.5min	23.42	23.88	6.1	10.74	<.001	<.001	-	0.149
	ACS-2min	23.70	24.13	6.35	10.54		<.001	0.149	-
SNR-IPFP									
	PI	8.05	7.93	1.21	1.79	<.001	-	<.001	<.001
	ACS-3.5min	8.94	8.81	1.47	2.2		<.001	-	<.001
	ACS-2min	7.08	7.09	1.43	2.18		<.001	<.001	-

Note. SD: standard deviation; IQR: interquartile range; PI: parallel acquisition technique; ACS: AI-assisted compressed sensing; ERD: edge rise distance; SNR: signal-to-noise ratio.

Table S3. Quantitative evaluation of edge rise distance (ERD) and signal-to-noise ratio (SNR) in sagittal PDFS images

Variable and protocol		Mean	Median	SD	IQR	*P* value for Friedman test	*P* value for post hoc analysis after Friedman test		
							PI	ACS-3.5min	ACS-2min
ERD									
	PI	0.85	0.77	0.25	0.25		-	NA	NA
	ACS-3.5min	1.09	0.74	0.86	0.59	0.218	NA	-	NA
	ACS-2min	0.83	0.78	0.24	0.23		NA	NA	-
SNR-Bone									
	PI	8.67	8.67	1.35	2.43	<.001	-	<.001	<.001
	ACS-3.5min	11.17	11.3	1.62	2.29		<.001	-	<.001
	ACS-2min	15.25	15.25	2.04	2.87		<.001	<.001	-
SNR-IPFP									
	PI	5.65	5.48	1.10	1.83		-	<.001	<.001
	ACS-3.5min	7.67	7.57	1.39	1.92	<.001	<.001	-	<.001
	ACS-2min	6.39	6.28	1.11	1.91		<.001	<.001	-

Note. PDFS: proton density-weighted fat suppression images; SD: standard deviation; IQR: interquartile range; PI: parallel acquisition technique; ACS: AI-assisted compressed sensing; ERD: edge rise distance; SNR: signal-to-noise ratio.

Table S4. Quantitative evaluation of edge rise distance (ERD) and signal-to-noise ratio (SNR) in transverse PDFS images

Variable and protocol	Mean	Median	SD	IQR	*P* value for Friedman test	*P* value for post hoc analysis after Friedman test		
						PI	ACS-3.5min	ACS-2min
ERD								
PI	0.91	0.79	0.33	0.32		-	<.001	<.001
ACS-3.5min	0.70	0.68	0.16	0.19	<.001	<.001	-	0.085
ACS-2min	0.75	0.72	0.17	0.22		<.001	0.085	-
SNR-Bone								
PI	8.24	8.2	0.98	1.71		-	<.001	<.001
ACS-3.5min	9.98	9.96	1.19	1.97	<.001	<.001	-	1.000
ACS-2min	9.88	9.35	1.25	1.89		<.001	1.000	-
SNR-IPFP								
PI	8.61	8.58	1.85	2.68	<.001	-	<.001	<.001
ACS-3.5min	10.24	10.21	2.37	3.52		<.001	-	<.001
ACS-2min	10.47	10.51	2.32	3.64		<.001	<.001	-

Note. PDFS: proton density-weighted fat suppression images; SD: standard deviation; IQR: interquartile range; PI: parallel acquisition technique; ACS: AI-assisted compressed sensing; ERD: edge rise distance; SNR: signal-to-noise ratio.

Table S5. Quantitative evaluation of edge rise distance (ERD) and signal-to-noise ratio (SNR) in coronal PDFS images

Variable and protocol		Mean	Median	SD	IQR	*P* value for Friedman test	*P* value for post hoc analysis after Friedman test		
							PI	ACS-3.5min	ACS-2min
ERD									
	PI	1.09	0.90	0.52	0.72	0.002	-	0.004	1.000
	ACS-3.5min	0.94	0.82	0.43	0.41		0.004	-	0.014
	ACS-2min	0.96	0.78	0.22	0.46		1.000	0.014	-
SNR-Bone									
	PI	8.45	8.47	0.88	1.51		-	<.001	<.001
	ACS-3.5min	9.74	9.73	1.00	1.50	<.001	<.001	-	<.001
	ACS-2min	11.62	11.72	1.13	1.61		<.001	<.001	-
SNR-IPFP									
	PI	6.42	4.44	2.10	4.05	<.001	-	<.001	<.001
	ACS-3.5min	8.52	8.63	2.30	3.28		<.001	-	<.001
	ACS-2min	11.21	10.90	3.11	4.62		<.001	<.001	-

Note. PDFS: proton density-weighted fat suppression images; SD: standard deviation; IQR: interquartile range; PI: parallel acquisition technique; ACS: AI-assisted compressed sensing; ERD: edge rise distance; SNR: signal-to-noise ratio.
